# Comparison of oxcarbazepine metabolite concentrations measured by EMIT-based Siemens Viva-ProE^®^ system and LC-MS/MS in Chinese patients

**DOI:** 10.3389/fphar.2025.1697679

**Published:** 2025-10-14

**Authors:** Ming Chen, Rong-Qi Lin, Yun-Yi Mao, Ying-Bin Huang, Jun-Nan Wu, Xue-Yong Li, Xue-Mei Wu, Yu Cheng, Hong-Qiang Qiu

**Affiliations:** ^1^ Department of Pharmacy, Fujian Medical University Union Hospital, Fuzhou, China; ^2^ College of Pharmacy, Fujian Medical University, Fuzhou, China; ^3^ Department of Pharmacy, Shanghang County Hospital, Shanghang, China

**Keywords:** oxcarbazepine, 10-hydroxycarbazepine, LC-MS/MS, EMIT, Viva-ProE^®^

## Abstract

**Background:**

Therapeutic drug monitoring (TDM) of oxcarbazepine’s active metabolite, the monohydroxy derivative (MHD), is essential for effective seizure management. Although liquid chromatography-tandem mass spectrometry (LC-MS/MS) is considered the gold standard for MHD quantification, its technical complexity restricts widespread clinical utility. The Siemens Viva-ProE^®^ System (SVPS), an automated immunoassay platform, presents a promising alternative. However, its comparability with LC-MS/MS warrants thorough and systematic evaluation.

**Objectives:**

This study established and validated an LC-MS/MS method for quantifying MHD in plasma and assessed the correlation and concordance of SVPS measurements using concentration-specific Deming regression. The objective was to evaluate the feasibility of replacing LC-MS/MS with SVPS for TDM LC-MS/MS in clinical practice.

**Methods:**

A validated LC-MS/MS method (linear range: 0.18–39.30 μg/mL; intra/inter-day RSD < 15%) and SVPS (measurable range: 0.00–50.00 μg/mL) were applied to analyze 158 plasma samples. Correlation and concordance between the methods were assessed using Spearman’s correlation, intraclass correlation coefficient (ICC), linear regression and Deming regression, Bland–Altman analysis, and Wilcoxon signed-rank tests. Stratified subgroup analyses, classified as low (<12 μg/mL), medium (12–22 μg/mL), and high (>22 μg/mL) concentration ranges, were conducted to evaluate the clinical acceptability of corrected SVPS values.

**Results:**

SVPS demonstrated a concentration-dependent positive bias (+13.04%) relative to LC-MS/MS. Despite this bias, strong overall correlation and concordance were observed (*r* = 0.9547, ICC = 0.952; *p* < 0.001). The overall Deming regression was defined by the equation: [LC-MS/MS] = 0.9763 × [SVPS] – 1.336. After correction, SVPS exhibited clinically acceptable concordance with LC-MS/MS within the low and medium concentration ranges, but not at higher concentrations.

**Conclusion:**

While uncorrected SVPS results exhibit a systematic bias that produces direct interchangeability with LC-MS/MS, applying a concentration-specific Deming correction enables clinically reliable TDM of MHD at concentrations below 22 μg/mL. However, method optimization is still required for accurate quantification in the high-concentration range.

## 1 Introduction

**FIGURE 1:**
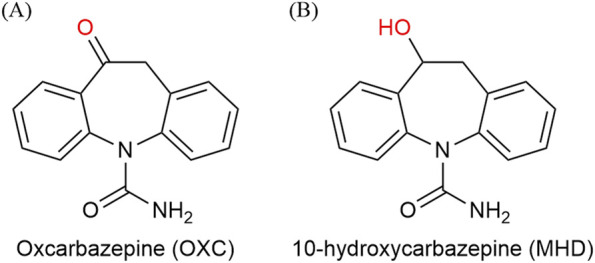
Chemical structures of **(A)** Oxcarbazepine and **(B)** 10-hydroxycarbazepine.

Epilepsy is a common chronic neurological disorder that is primarily managed with pharmacotherapy (Perucca, 2021; Abou-Khalil, 2025). Oxcarbazepine (OXC), a second-generation antiseizure medications (ASMs) (Figure 1A), is widely used in the treatment of partial seizures. Its therapeutic effects are largely mediated through its active metabolite, the monohydroxy derivative (MHD) (Figure 1B) (Flesch, 2004; Kośmider et al., 2023). Therapeutic drug monitoring (TDM) plays a pivotal role in neuropsychopharmacology by guiding individualized treatment strategies for patients receiving antiseizure medications. Recent real-world studies have shown that TDM significantly improves seizure control, reduces dose-related toxicity, and supports clinical decision-making in both epilepsy and neuropsychiatric populations (Biso et al., 2023; Lim et al., 2024). Due to the considerable interindividual variability in pharmacokinetics and its therapeutic window (commonly 3–35 μg/mL) (Patsalos et al., 2008), current clinical guidelines recommend TDM to optimize efficacy, minimize adverse effects (e.g., somnolence, ataxia, hyponatremia), and enhance treatment adherence (Nedelman et al., 1999; Barcs et al., 2000; Sachdeo et al., 2001; Striano et al., 2006; Sattler et al., 2015).

Liquid chromatography-tandem mass spectrometry (LC-MS/MS) is regarded as the gold standard for quantifying MHD owing to its high sensitivity and specificity (Patsalos et al., 2018). However, its widespread clinical use is limited by high operational cost, technical complexity, and time-consuming sample preparation (Grebe and Singh, 2011). In contrast, immunoassay-based techniques such as enzyme-multiplied immunoassay technique (EMIT), chemiluminescent immunoassay (CLIA), and microparticle enzyme immunoassay (MEIA) provide rapid, automated alternatives with streamlined workflows (Sami Shaikh et al., 2017). The Siemens Viva-ProE^®^ System (SVPS), an EMIT-based platform, enables high-throughput analysis with minimal manual intervention. Nevertheless, concerns remain regarding limitations in analytical specificity and potential cross-reactivity, which may affect measurement accuracy (Hughey and Colby, 2019; Sotnikov et al., 2021).

While previous studies have compared immunoassays with chromatographic methods for monitoring ASMs, no research to date has specifically evaluated SVPS against LC-MS/MS for MHD quantification. More importantly, no study has systematically investigated concentration-dependent bias or developed correction strategies to improve the interchangeability between SVPS and LC-MS/MS. A thorough understanding of SVPS performance across various MHD concentration ranges is critical to ensure its reliability in clinical practice.

Therefore, this study was designed to systematically evaluate the correlation, concordance, and concentration-dependent bias between SVPS and LC-MS/MS across therapeutic subranges. A further aim was to develop concentration-specific Deming correction models to enhance the agreement between the two methods and to assess corrected SVPS results could serve as a reliable substitute for LC-MS/MS in clinical TDM. Additionally, the study sought to validate a robust LC-MS/MS method for the quantification of MHD in human plasma.

## 2 Materials and methods

### 2.1 Chemicals, reagents, and materials

The reference standard for MHD (purity: 96%) and the internal standard (IS), MHD-d4 (purity: 95%), were obtained from HOPE PRECISION MEDICINE Inc. (Hangzhou, China). EMIT^®^ 2000 Oxcarbazepine Metabolite Calibrators (Lot: 20,240,504; expiry date: 20,250,918) and EMIT^®^ 2000 Oxcarbazepine Metabolite Test Kit (Lot: 20,240,504; expiry date: 20251119) were purchased from Siemens Healthcare Diagnostics Ltd. (Newark, NJ, United States). Methanol (HPLC grade) and formic acid (LC-MS grade) were obtained from Merck KGaA (Darmstadt, Germany). Ultrapure water was prepared using a Milli-Q water purification system (Millipore, Bedford, MA, United States of America).

Cryopreserved human plasma samples used in this study were surplus specimens obtained from the clinical laboratory of Fujian Medical University Union Hospital (Fuzhou, China). The study protocol was conducted in accordance with the principles of the Declaration of Helsinki and was approved by the institutional ethics committee (approval number: 2020KY004).

### 2.2 Validated of LC-MS/MS analysis

#### 2.2.1 Instrumentation and analytical conditions

LC-MS/MS analysis was carried out using a Shimadzu Jasper™ HPLC system (Kyoto, Japan) coupled to an AB SCIEX Triple Quad™ 4500MD mass spectrometer (Singapore). Separation was achieved on a Shim-pack GSP-HP C18 column (50 × 2.1 mm, 3 µm) maintained at 40 °C.

The mobile phase consisted of 0.1% formic acid in water (A) and methanol (B), with the following gradient elution:0–1.0 min, 90% A; 1.0–1.9 min, 60% B; 1.9–2.5 min, 60% B; 2.5–3.0 min, 90% A. The flow rate was 0.4 mL/min, and the total run time was 3.0 min. The LC flow was directed to the MS detector between 1.0 and 3.0 min.

The electrospray ionization (ESI) source was operated in positive-ion mode with an ion spray voltage of 5.5 kV. The source temperature was 450 °C. Curtain gas, ion source gas 1, and gas 2 were set at 35, 40, and 40 psi, respectively. Multiple reaction monitoring (MRM) was employed to monitor transitions of m/z 255.1 → 194.0 for MHD and m/z 259.1 → 198.2 for the IS.

#### 2.2.2 Preparation of solutions, calibration standards, and quality control samples

Stock solutions of MHD and IS (1.00 mg/mL) were prepared in methanol and stored at −20 °C. Working solutions were prepared by diluting the stock solutions with methanol.

Calibration standards were prepared by spiking blank plasma with working solutions to yield concentrations of 0.18, 0.38, 1.00, 2.02, 3.98, 10.06, 20.06, and 39.30 μg/mL.

Quality control (QC) samples were prepared at four concentration levels: 0.18 μg/mL (lower limit of quantification, LLOQ), 7.57 μg/mL (low QC, LQC), 15.12 μg/mL (medium QC, MQC), and 30.25 μg/mL (high QC, HQC).

#### 2.2.3 Sample preparation

A 25 µL aliquot of plasma was combined with 100 µL of IS working solution (1.00 mg/mL) in a 2.0 mL Eppendorf tube. The mixture was vortexed for 1 min and centrifuged at 13,000 rpm for 5 min. Then, 10 µL of the supernatant was transferred to a new tube and diluted with 490 µL of purified water. After vortexing for 30 s and recentrifugation under the same conditions, a 100 µL aliquot of the final solution was injected into the LC-MS/MS system for analysis.

#### 2.2.4 Method validation

The LC-MS/MS method was validated following EMA and FDA guidelines (Agency, 2011; Food and Administration, 2018), including assessment of selectivity, linearity, LLOQ, intra- and inter-day accuracy and precision, recovery, matrix effects, carryover, and analyte stability under various storage and processing conditions. The method exhibited acceptable performance with a linear calibration range from 0.18 to 39.30 μg/mL (*r* ^*2*^ > 0.99), accuracy and precision within ± 15% across QC levels, and no significant interferences observed in blank samples.

### 2.3 Enzyme multiplied immunoassay technique assay performance

The plasma concentration of MHD was determined using an automated enzyme immunoassay analyzer (Siemens Viva-ProE^®^ System, Germany), following the manufacturer’s instructions for the EMIT^®^ 2000 Oxcarbazepine Metabolite Assay. The plasma samples were vortexed for 1 min and subsequently centrifuged at 13,000 rpm for 5 min. The resulting supernatant was collected and used for subsequent analysis.

Calibration standards were prepared at concentrations of 0.00, 2.00, 5.00, 12.00, 25.00, and 50.00 μg/mL. QC samples at three concentration levels were incorporated into each analytical batch.

### 2.4 Comparison of EMIT and LC-MS/MS

According to previous literature (Bouquié et al., 2010), the clinically effective plasma concentration of MHD genernally ranges from 12 to 30 μg/mL, with a significantly increased risk of adverse effects when concentrations exceed 30 μg/mL (Striano et al., 2006; Patsalos et al., 2008). Based on clinical guidelines and the actural distribution of measured concentrations, the 128 plasma samples were stratified into three subgroups: <12.0 μg/mL (low, below the therapeutic range), 12.0–22.0 μg/mL (medium, covering the lower to mid-therapeutic range), and >22.0 μg/mL (high, encompassing the upper therapeutic range and near-toxic levels). This stratification scheme was designed to reflect clinically meaningful concentration categories while ensuring adequate sample sizes in each subgroup for robust statistical comparison.

The initial SVPS concentrations, as well as those corrected using the Deming regression equation, were compared against the LC-MS/MS results to evaluate inter-measurement differences. The relative prediction error (PE, Equation 1) was calculated using the corrected SVPS values. Bias was accessed via the mean prediction error (MPE, Equation 2), while absolute accuracy was evaluated using the root mean square prediction error (RMSE, Equation 3). Clinical acceptability was defined based on pre-established thresholds: MPE within ± 15% for and RMSE below 15%–20%.

To validate the performance of the Deming regression correction, a separate set of 30 randomly selected samples was used. The absolute errors (in µg/mL) between the corrected SVPS concentrations and the reference LC-MS/MS results was visualized using box plots stratified by concentration subgroup. Analytical performance was assessed by calculating MPE, RMSE, Pearson’s correlation coefficient (r), and the intraclass correlation coefficient (ICC).

$$PE=\frac{{\left[SVPS\right]}_{corrected}-\left[LC-MS/MS\right]}{\left[LC-MS/MS\right]}\times 100\%$$ (1)

$$MPE=\frac{1}{N}\sum \left({PE}_{i}\right)$$ (2)

$$RMSE=\sqrt{\frac{1}{N}\sum {\left({PE}_{i}\right)}^{2}}$$ (3)

### 2.5 Statistical analysis

The method comparison study was designed and performed according to the key recommendations of the Clinical and Laboratory Standards Institute (CLSI) EP09-A3 guideline. All statistical analyses were performed using GraphPad Prism (version 10.1.0; GraphPad Software, CA, United States) and the R software environment (version 4.3.2; R Foundation for Statistical Computing, Vienna, Austria). Continuous data are presented as mean ± standard deviation (SD) or median (range), based on their distribution. Normality was evaluated using the Kolmogorov-Smirnov test and the D’Agostino-Pearson test. Correlation between the two methods was assessed through linear regression, Deming regression, Spearman’s correlation, and the intraclass correlation coefficient (ICC). Method concordance was evaluated using Wilcoxon signed-rank tests and Bland-Altman analysis to quantify absolute and relative biases. Fisher’s exact test was employed to examine associations between categorical outcomes of the methods, where a significant result indicates correlation. In contrast, McNemar’s test was applied to evaluate discordance; a significant p-value suggests a lack of agreement between methods (Balakrishnan, 2014; Smeltzer and Ray, 2022).

## 3 Results

### 3.1 Basic information and concentration distribution of plasma samples

A total of 160 plasma samples were analyzed using both the SVPS and LC-MS/MS methods. Two samples with concentrations below the LLOQ and were consequently excluded from statistical analysis. The remaining 158 samples were utilized for descriptive statistics and concentration distribution analysis. To ensure a robust evaluation, the dataset was then divided into a method comparison cohort (n = 128) for establishing the Deming regression equation and an independent validation cohort (n = 30) for verifying the correction performance. The age of the patients from whom samples were obtained ranged from 0.3 to 72 years, with a median age of 16 years. Additional demographic characteristics are summarized in Table 1.

**TABLE 1:** Baseline demographic and clinical characteristics of the patients (N = 158).

Characters (unit)	Mean ± SD	Median (range)
Age (Years)	20.7 ± 14.9	16.0 (0.3–72)
Gender (Male/Female)	92/66	—
MHD concentration (µg/mL)	17.0 ± 6.9	17.1 (1.7–33.0)
Diagnosis, N (%)	Epilepsy, 158	—
Total bilirubin (TBil, µmol/L)	7.2 ± 4.3	6.3 (2.8–43.0)
Direct bilirubin (DBil, µmol/L)	1.6 ± 1.2	1.4 (0.2–13.0)
Indirect bilirubin (IBil, µmol/L)	5.3 ± 2.2	4.9 (0.3–12.5)
Total protein (TP) (g/L)	67.1 ± 15.7	70.2 (0.5–82.5)
Albumin (ALB, g/L)	45.1 ± 3.2	45.5 (32.8–52.8)
Globulin (GLB, g/L)	25.0 ± 5.3	25.6 (2.1–36.8)
ALT (U/L)	20.9 ± 25.9	15.0 (1.0–276)
AST (U/L)	24.1 ± 10.9	22.0 (1.0–86.0)
Creatinine (CREA, µmol/L)	55.1 ± 18.3	55.5 (4.0–109.0)
Uric acid (UA, µmol/L)	301.7 ± 121.8	294.5 (19.0–798.0)
Na^+^ (mmol/L)	132.7 ± 28.9	140.1 (10.9–145.6)
K^+^ (mmol/L)	4.2 ± 0.4	4.2 (3.5–5.7)
Red blood cell count (RBC) (10^12^/L)	4.6 ± 0.6	4.6 (0.9–6.2)
Hemoglobin (HGB) (g/L)	131.7 ± 25.6	134.0 (10.0–176.0)
Platelet (PLT) (10^9^/L)	273.8 ± 74.1	262.0 (147.0–599.0)

Data are expressed as Mean ± SD, and Median (range) except for gender and Diagnosis.

Among the 158 included samples, MHD concentrations measured by SVPS ranged from 2.1 to 33.84 μg/mL (mean ± SD: 15.4 ± 6.0 μg/mL; median: 15.0 μg/mL), whereas concentrations determined by LC-MS/MS ranged from 1.94 to 32.42 μg/mL (mean ± SD: 13.7 ± 5.9 μg/mL; median: 12.5 μg/mL). On average, SVPS yielded MHD concentrations that were 12.4% higher than those obtained by LC-MS/MS. The overall concentration distributions for both methods are presented in Table 2.

**TABLE 2:** The distribution of total MHD sample concentrations measured by the SVPS and LC-MS/MS methods.

Concentration distribution (µg/mL)		SVPS (n%)			Total
		<12.0	12.0–22.0	>22.0	
LC-MS/MS (n%)	< 12.0	50 (31.6%)	18 (11.4%)	0 (0%)	68 (43.0%)
	12.0–22.0	0 (0.0%)	69 (43.7%)	9 (5.7%)	78 (49.4%)
	>22.0	0 (0.0%)	0 (0.0%)	12 (7.6%)	12 (7.6%)
Total		50 (31.6%)	87 (55.1%)	21 (13.3%)	158 (100%)

### 3.2 LC-MS/MS method validation

#### 3.2.1 Selectivity

**FIGURE 2:**
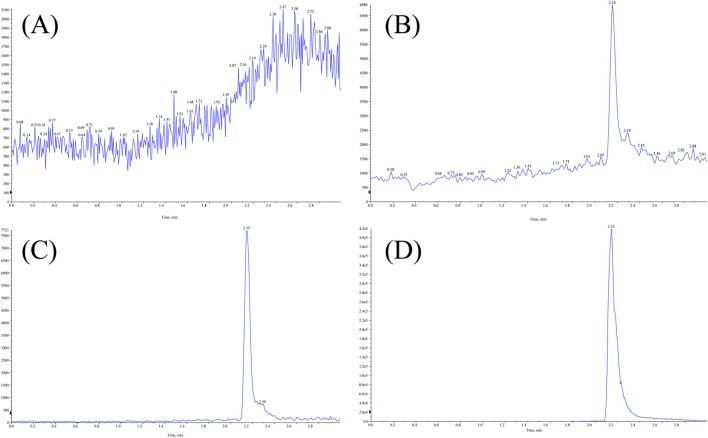
Representative LC-MS/MS chromatograms of MHD and IS in human plasma: **(A)** blank plasma; **(B)** LLOQ; **(C)** IS; **(D)** patient sample.

The retention times for MHD and IS were 2.16 min and 2.15 min, respectively. No interfering peaks from endogenous plasma components were detected at the corresponding retention times, confirming high method selectivity. Representative chromatograms of blank plasma, LLOQ, IS, and a patient sample are presented in Figure 2.

#### 3.2.2 Linearity and lower limit of quantification (LLOQ)

**FIGURE 3:**
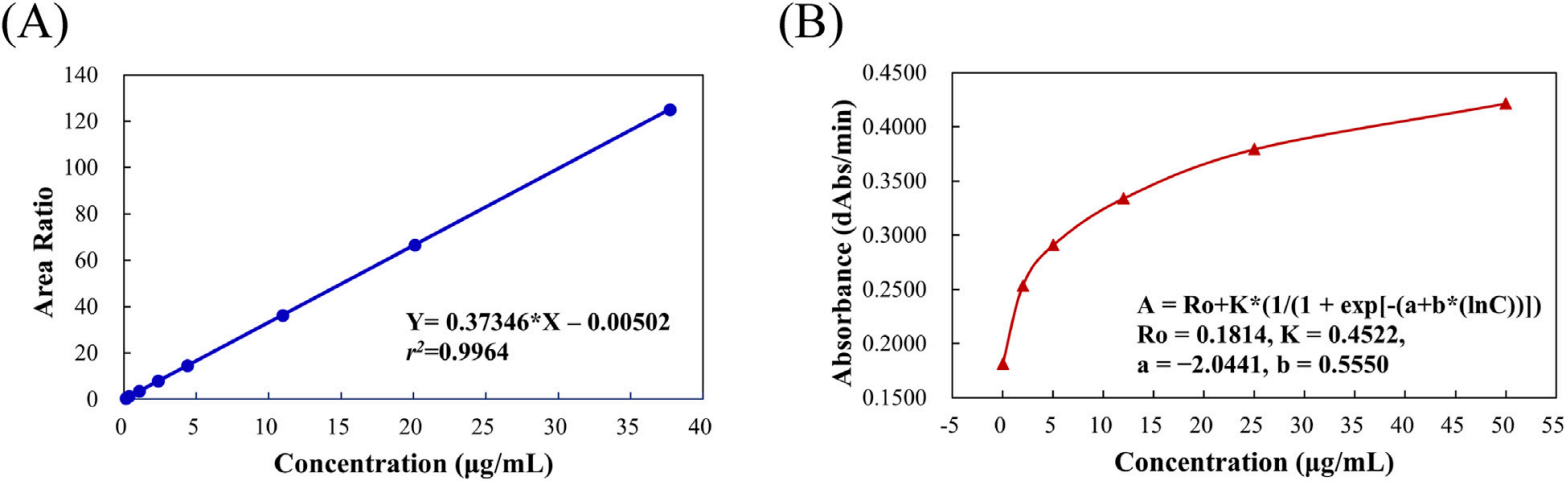
Representative calibration curves for MHD quantification using **(A)** LC-MS/MS (0.18–39.30 μg/mL) and **(B)** Viva-ProE^®^ (0.00–50.00 μg/mL).

A calibration curve for MHD was established over the concentration range of 0.18–39.30 μg/mL. The weighted (1/x^2^) linear regression equation was y = 0.37346x – 0.00502 (*r* ^*2*^ = 0.9964) (Figure 3A), indicating excellent linearity. The LLOQ was determined to be 0.18 μg/mL, supported by a signal-to-noise ratio exceeding 5:1.

#### 3.2.3 Accuracy and precision

The intra-day and inter-day accuracy and precision for MHD quantification are summarized in Table 3. All results met pre-defined acceptance criteria, with RE and RSD within ± 20% for LLOQ QC level and within ± 15% for low, medium, and high QC levels.

**TABLE 3:** Intra-day and inter-day precision and accuracy for MHD in human plasma samples.

QC level	Intra-day (n = 6)		Inter-day (n = 6 × 3)	
	A	P	A	P
LLOQ	14.41	10.85	12.09	16.29
LQC	4.48	8.88	11.73	11.47
MQC	10.19	5.58	7.74	14.37
HQC	11.80	5.73	13.89	13.96

A, accuracy and data are expressed as RE (%); P, precision, and data are expressed as RSD (%).

#### 3.2.4 Stability

The stability of MHD in human plasma was evaluated under four different storage conditions (see Table 4 for details). The analyte was found to be stable across all evaluated scenarios, with both RE% and RSD% values meeting the predefined acceptance criteria of ± 15%.

**TABLE 4:** Stability performance of MHD under specified storage conditions.

Storage condition	QC level	RE (%)	RSD (%)
Room temperature (25 °C, 24 h)	LQC	6.22	6.08
	HQC	14.01	4.36
Auto-sampler stability (4 °C, 24 h)	LQC	0.88	7.67
	HQC	7.26	8.93
Freeze-thaw stability (−20 °C, three cycles)	LQC	14.89	5.40
	HQC	7.07	4.49
Long-term stability (−80 °C, 28days)	LQC	11.46	3.56
	HQC	7.38	2.99

#### 3.2.5 Recovery, matrix effects, and carryover

The recovery and matrix effect were evaluated at three QC levels (7.57, 15.12, and 30.25 μg/mL) using six different lots of blank human plasma. The mean extraction recovery of MHD ranged from 94.1% ± 4.4% to 96.3% ± 3.8% with RSDs ≤ 5.2%, indicating high and consistent extraction efficiency. The IS-normalized matrix factors showed excellent precision, with RSDs ranging from 3.4% to 4.5%, which demonstrates that matrix effects are negligible and do not interfere with the quantitative accuracy of the method.

Carryover was evaluated by injecting a blank sample after an upper limit of quantification sample. The carryover was found to be 8.5% of the LLOQ for MHD and less than 5.0% for the IS, which is significantly below the 20% threshold, indicating the carryover was deemed negligible for MHD and IS during the chromatographic determination.

### 3.3 SVPS assay

A four-point logarithmic curve was used to obtain the formula for the calibration curve between 0.00 and 50.00 μg/mL. And the formula was as follows: A = Ro + K* (1/(1 + exp [-(a + b* (lnC))])); where Ro = 0.1814, K = 0.4522, a = −2.0441, and b = 0.5550. Analyte concentrations were determined using the regression equation shown in Figure 3B.

### 3.4 Correlation and concordance between LC-MS/MS and SVPS assays

#### 3.4.1 Total correlation and concordance analysis

**FIGURE 4:**
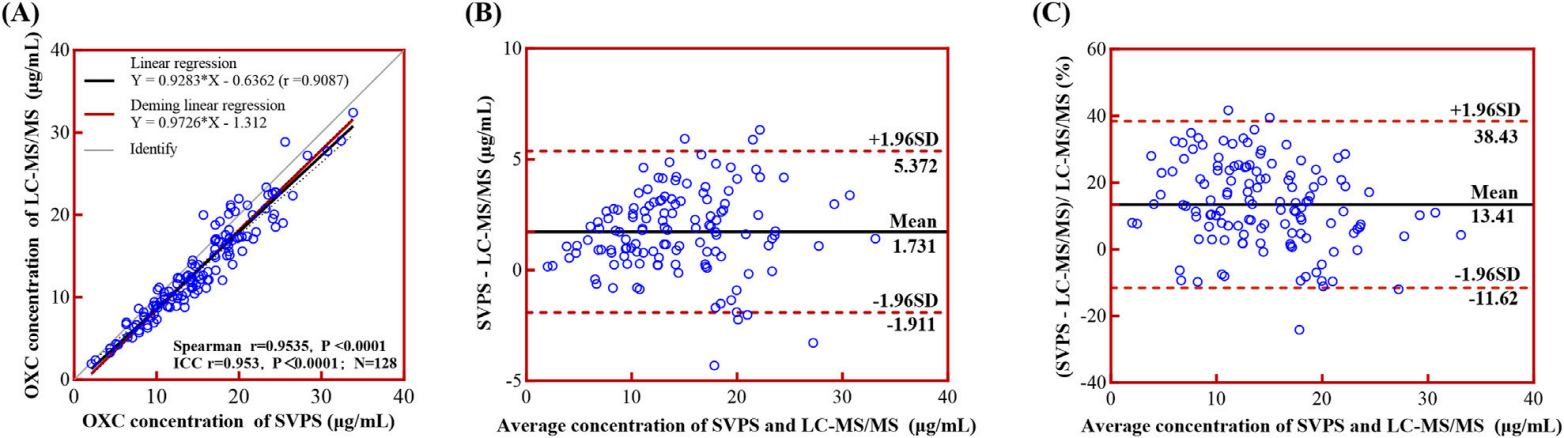
Linear regression and Bland-Altman analyses comparing LC-MS/MS and SVPS assays for MHD concentration. *Note:* **(A)** Simple linear regression (black solid line with 95% CI as black dotted line) and Deming regression (red solid line) curves. **(B)** Bland-Altman plot of absolute bias. **(C)** Bland-Altman plot of percentage bias. In Bland-Altman plots **(B,C)**, the mean bias is shown as a black solid line and the ±95% LoA as red dotted lines.

The concentrations of MHD measured by SVPS and LC-MS/MS were found to be non-normally distributed, as evidenced by the Kolmogorov–Smirnov and D’Agostino–Pearson tests (*p* < 0.001). Spearman’s correlation and ICC analyses demonstrated a strong agreement between the two methods (r = 0.9547, ICC = 0.952; *p* < 0.001). Linear regression analysis (Figure 4A) yielded an ordinary least squares (OLS) equation of [LC-MS/MS] = 0.9313 × [SVPS] – 0.6429 (*r* ^*2*^ = 0.9076). Deming regression produced a comparable model: [LC-MS/MS] = 0.9763 × [SVPS] – 1.336.

Regarding method concordance, the paired Wilcoxon test indicated a statistically significant difference between the two methods (*p* < 0.0001), suggesting poor overall concordance. Bland–Altman analysis (Figures 4B,C) further confirmed this discordance, revealing a mean absolute bias of 1.70 μg/mL (95% limits of concordance (LoA): −1.88–5.28 μg/mL) and a mean relative bias of 13.04% (95% LoA: −11.26%–37.34%). These results indicate that SVPS systematically overestimated MHD concentrations relative to LC-MS/MS, although the magnitude of this bias falls within acceptable analytical limits.

#### 3.4.2 Correlation and concordance analysis in different concentration groups

**FIGURE 5:**
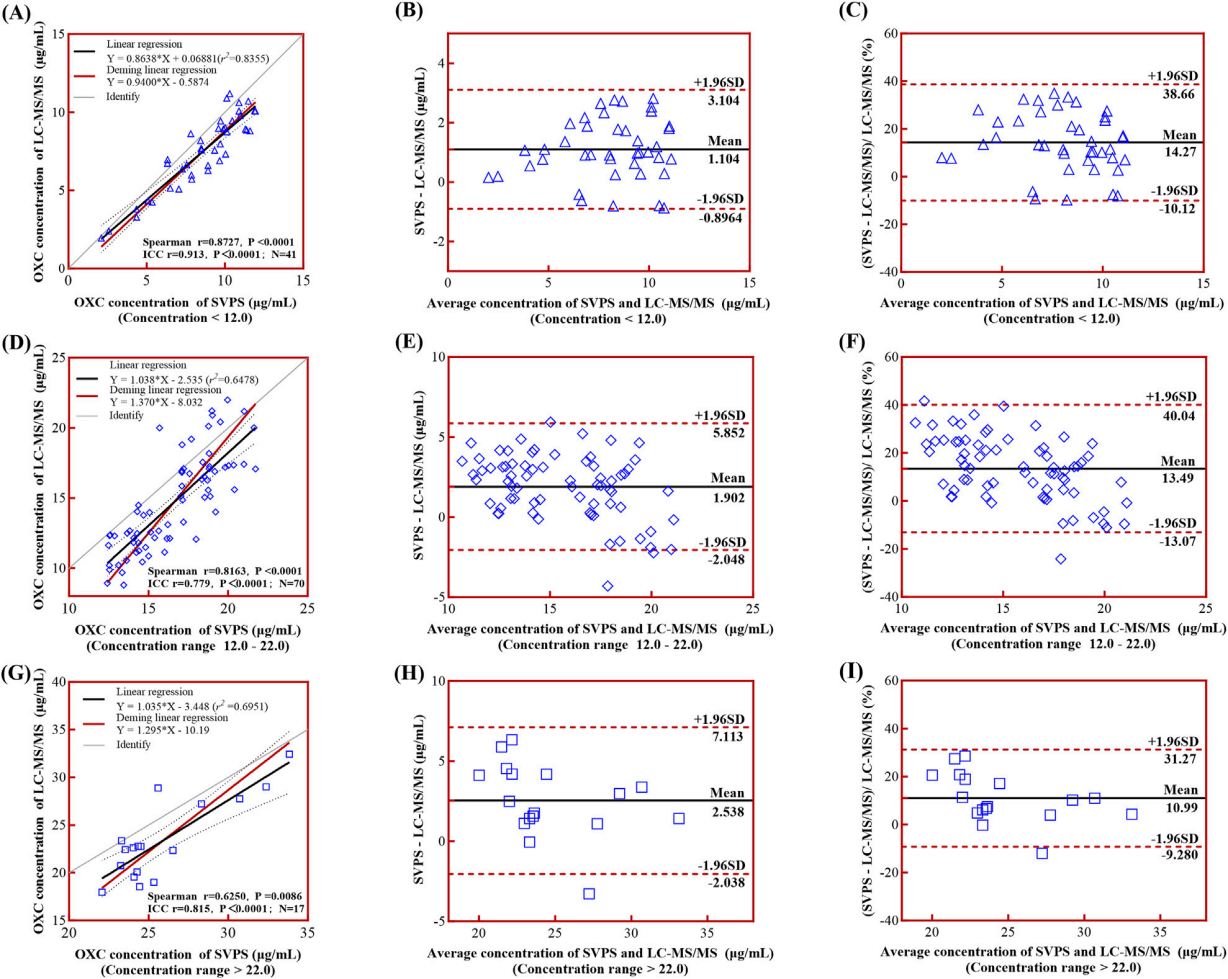
Linear regression and Bland-Altman analyses comparing LC-MS/MS and SVPS assays across concentration groups. *Note:* **(A)**, **(D)**, **(G)** Simple linear regression (black solid line) and Deming regression (red solid line) curves with 95% limits of agreement (LoA, black dotted lines) for low-, medium-, and high-concentration groups, respectively. **(B)**, **(E)**, **(H)** Bland-Altman plots of absolute biases. **(C)**, **(F)**, **(I)** Bland-Altman plots of percentage biases. In all Bland-Altman plots **(B,C,E,F,H,I)**, the mean bias is shown as a black solid line and the ±95% LoA as red dotted lines.

In the high-concentration group (>22.0 μg/mL), the correlation was weak, and internal concordance was moderate (Spearman r = 0.6250, p = 0.0086; ICC = 0.815, *p* < 0.001) (Figure 5A). In contrast, both the low-concentration (<12.0 μg/mL) and medium-concentration groups (12.0–22.0 μg/mL) demonstrated strong correlations and high internal concordance, with Spearman r = 0.8727, ICC = 0.913 for the low group, and Spearman r = 0.8163, ICC = 0.779 for the medium group (both *p* < 0.001; Figures 5D,G, respectively). The corresponding Deming regression equations for each concentration stratum are presented in Table 5.

**TABLE 5:** Deming regression formulas for MHD concentration stratified by these groups.

Concentration range by SVPS (µg/mL)	Deming regression formula	Spearman’s correlation	ICC
<12.0 (n = 41)	Y = 0.9400*X - 0.5874	0.8727, *p* < 0.0001	0.913, *p* < 0.0001
12.0–22.0 (n = 70)	Y = 1.370*X - 8.032	0.8163, *p* < 0.0001	0.779, *p* < 0.0001
>22.0 (n = 17)	Y = 1.295*X - 10.19	0.6250, *p* = 0.0086	0.815, *p* < 0.0001
ALL sample (n = 128)	Y = 0.9726*X - 1.312	0.9535, *p* < 0.0001	0.953, *p* < 0.0001

Bland-Altman analysis revealed a concentration-dependent trend in absolute bias, which increased progressively across concentration groups: low (1.10 μg/mL; 95% LoA: −0.90–3.10 μg/mL), medium (1.90 μg/mL; 95% LoA: −2.05–5.85 μg/mL), and high (2.54 μg/mL; 95% LoA: −2.04–7.11 μg/mL) (Figures 5B,E,H). In contrast, the relative bias decreased at higher concentrations: low (14.27%; 95% LoA: −10.12%–38.66%), medium (13.49%; 95% LoA: −13.07%–40.40%), and high (10.99%; 95% LoA: −9.28%–31.27%) (Figures 5C,F,I). This pattern is consistent with established diagnostic criteria for heteroscedasticity in Bland-Altman analysis, wherein absolute differences vary proportionally with the magnitude of the measurement (Altman and Bland, 1983).

### 3.5 Comparative evaluation of SVPS and LC-MS/MS methods and implications for clinical therapy

Given that SVPS consistently yielded higher MHD concentrations than LC-MS/MS, we sought to evaluate whether this measurement bias could influence clinical decision-making. Two critically relevant concentration thresholds were defined based on the observed distribution of measured values: 12 μg/mL and 22 μg/mL. Using LC-MS/MS as the reference method, we compared the classification agreement of original SVPS concentrations. Fisher’s exact test revealed a significant overall association between the two methods (*p* < 0.0001). However, McNemar’s test revealed significant classification discordance at both thresholds (*p* < 0.05), suggesting that direct substitution of uncorrected SVPS results for LC-MS/MS is not clinically acceptable.

**FIGURE 6:**
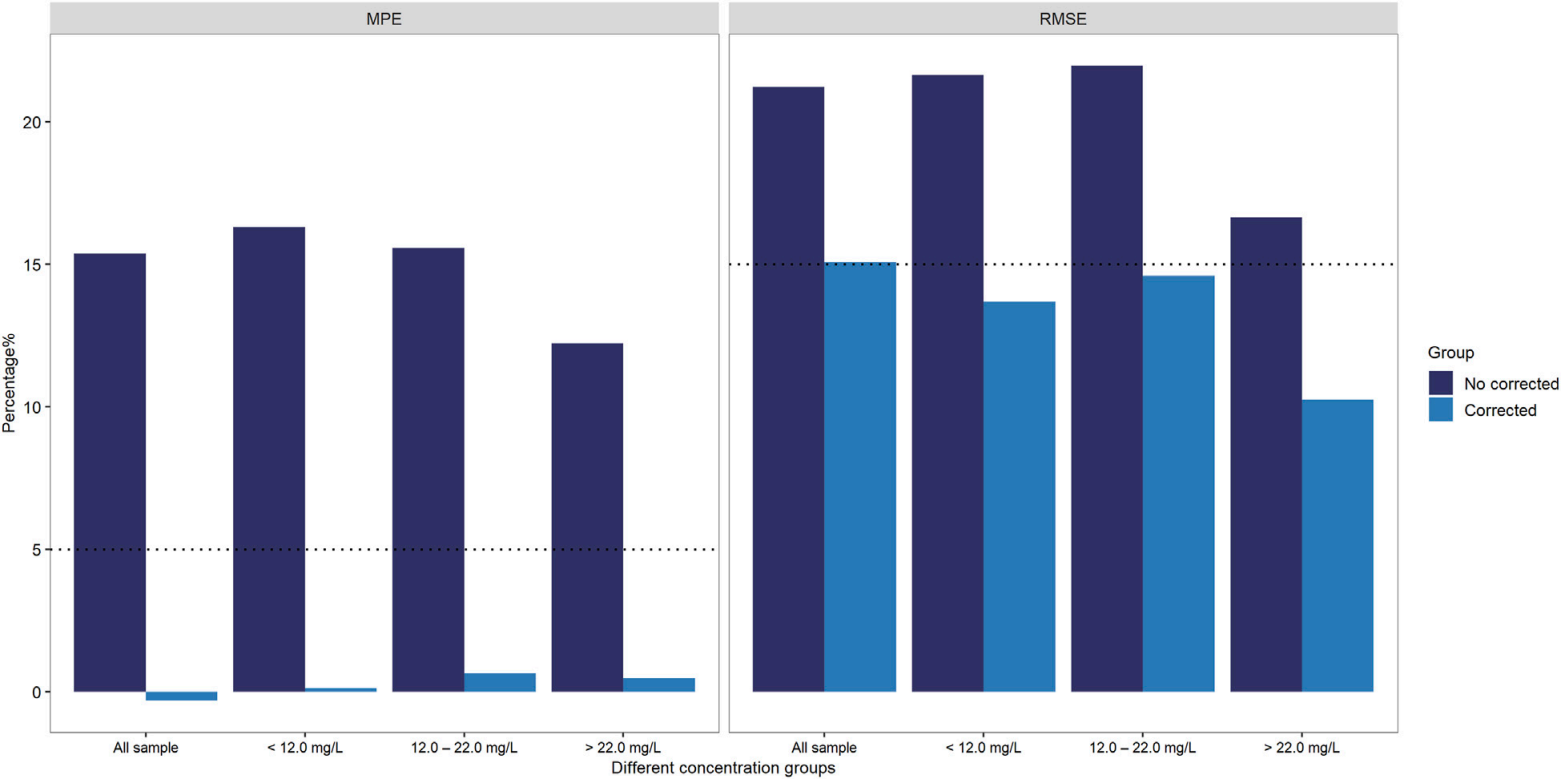
Accuracy improvement post-Deming regression: MPE and RMSE of SVPS vs. LC-MS/MS stratified by concentration groups.

**FIGURE 7:**
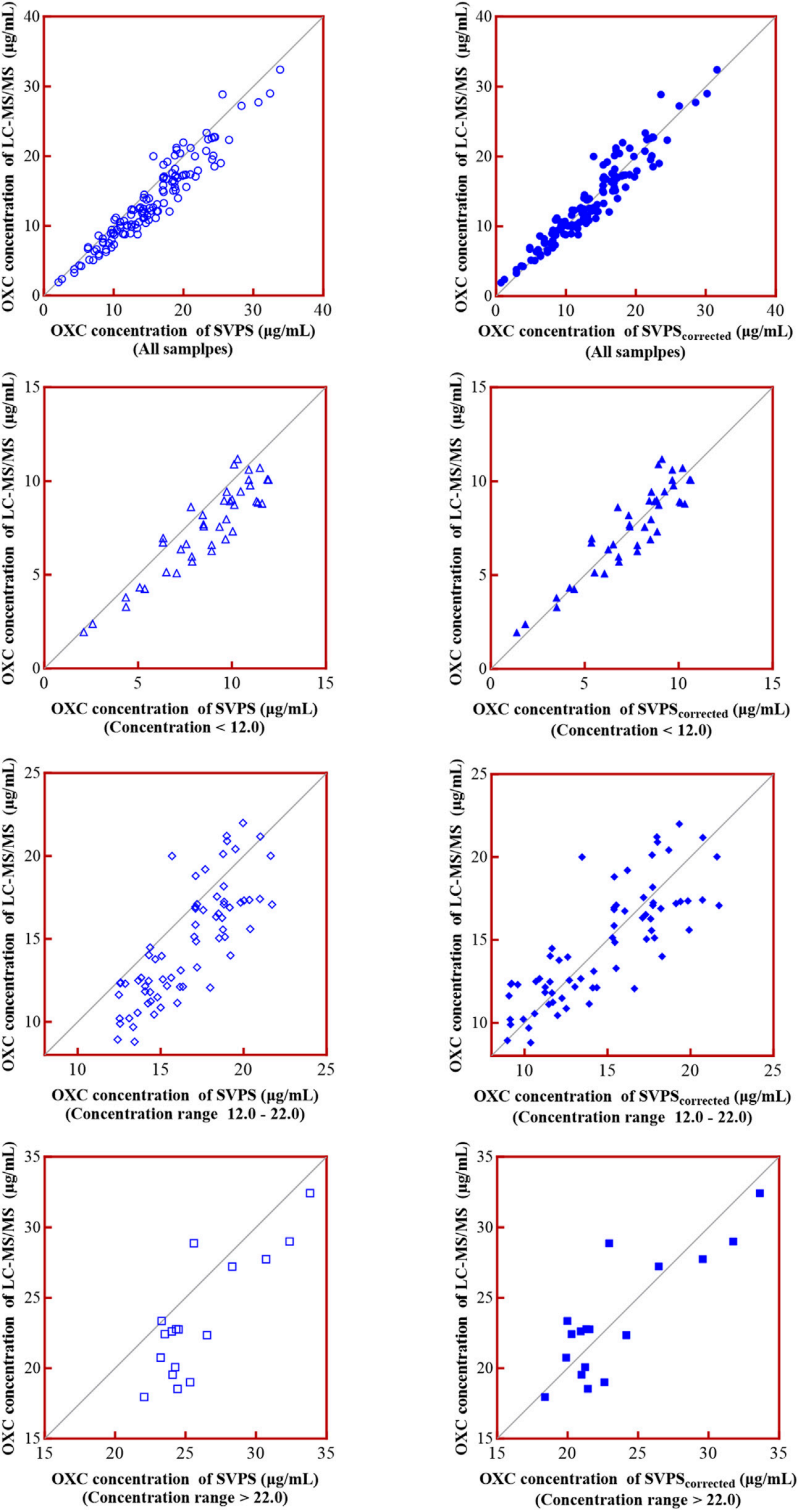
Goodness-of-fit of SVPS vs. LC-MS/MS before and after Deming regression correction.

To address the systematic bias of SVPS, we applied a Deming linear regression equation to adjust the SVPS results and re-evaluated their correlation and concordance with LC-MS/MS results. As summarized in Table 6, both original and Deming-adjusted SVPS concentrations showed significant correlation with LC-MS/MS (*p* < 0.0001). However, the original SVPS values exhibited significant classification discordance at both 12 μg/mL and 22 μg/mL thresholds according to McNemar’s test (*p* < 0.05). In contrast, after adjustment with Deming regression, McNemar’s test showed no significant disagreement at either thresholds (*p* > 0.05), indicating markedly improved concordance. This improvement was further supported by a reduction in error metrics: after correction, the MPE was consistently below 5% across all concentration groups, and the RMSE was generally within 15% for all ranges (Figure 6). Additionally, as illustrated in Figure 7, the corrected SVPS values showed substantially improved alignment with the identity line compared to the original values. These results suggest that Deming-adjusted SVPS values can serve as a reliable and clinically interchangeable alternative to LC-MS/MS in most scenarios. Nevertheless, confirmation by LC-MS/MS remains advisable in critical or equivocal clinical situations.

**TABLE 6:** Contingency tables used two alternative cut-off values to compare TDM-based indications by LC-MS/MS and SVPS or adjusted SVPS by deming linear regression.

“n” (for count)	MHD determined by the SVPS with cut-off at 12 μg/mL			Total	*p*-value	MHD determined by the SVPS and corrected by DLR with threshold at 12 μg/mL			Total	*p*-value
MHD determined by LC-MS/MS with a cutoff at 12 μg/mL		<12	≥12		*p* Fisher < 0.0001		<12	≥12		*p* Fisher < 0.0001
	<12	41	16	57		<12	49	8	57	
	≥12	0	71	71		≥12	4	67	71	
Total		41	87	128	*p* McNemar = 0.00018		53	75	128	*p* McNemar = 0.3865
	MHD determined by SVPS with cut-off at 22 ng/mL					MHD determined by SVPS and corrected by DLR with threshold at 22 μg/mL				
MHD determined by LC-MS/MS with a cutoff at 22 μg/mL		≤22	>22		*p* Fisher < 0.0001		≤22	>22		*p* Fisher < 0.0001
	≤22	111	6	117		≤22	113	4	117	
	>22	0	11	11		>22	2	9	11	
Total		111	17	128	*p* McNemar = 0.0412		115	13	128	*p* McNemar = 0.6831

DLR, dynamic linear regression.

### 3.6 Validation of the correction effect of deming regression correction

**FIGURE 8:**
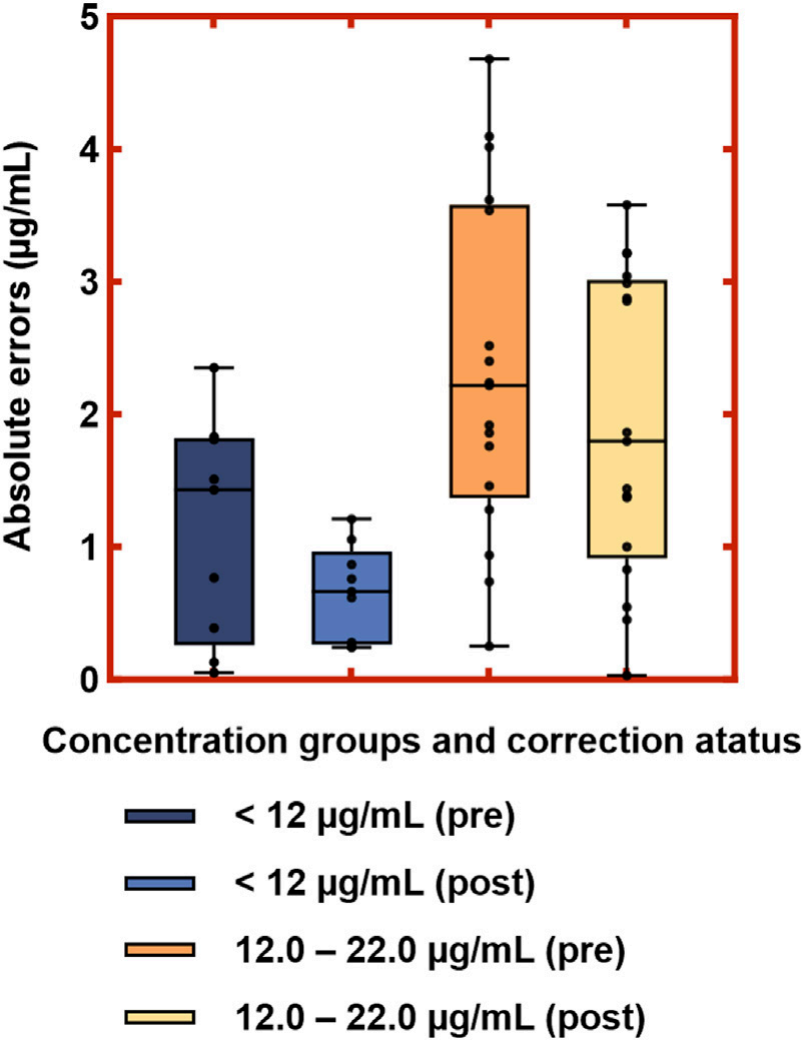
Distribution of absolute errors between Deming-corrected SVPS and LC-MS/MS concentrations in the low and medium concentration groups. *Note:* Boxes represent interquartile range (IQR); center lines indicate medians; whiskers show minimum and maximum values; dots represent individual data points.

Given the limited sample size and reduced clinical relevance in the high-concentration range, subsequent visual (Figure 8) and performance analyses (Table 7) were focused exclusively on the low- and medium-concentration groups. Figure 8 displays the distribution of absolute errors between Deming-corrected SVPS and LC-MS/MS values, calibrated using the group-specific regression equations provided in Table 5. The low-concentration group (<12.0 μg/mL) showed optimal correction performance, with tightly clustered errors (median absolute error < 1.0 μg/mL), indicating excellent concordance with the reference method. The medium-concentration group (12.0–22.0 μg/mL) exhibited moderately greater dispersion, though the variation remained within clinically acceptable limits. These findings demonstrate that concentration-specific Deming correction significantly improves the agreement between SVPS and LC-MS/MS within therapeutically relevant concentration ranges.

**TABLE 7:** Performance metrics of validation samples before and after deming regression correction in the low- and medium-concentration groups.

Concentration group (µg/mL)	Pre-correction		Post-correction		Mean Abs. Errors (µg/mL)	Spearman’s correlation	ICC
	MPE	RMSE	MPE	RMSE			
<12.0 (n = 9)	14.15%	17.32%	−0.85%	10.49%	0.66	0.8000,*p* = 0.0138	0.945, *p* < 0.0001
12.0–22.0 (n = 17)	12.77%	18.61%	0.82%	14.26%	1.91	0.8358,*p* < 0.0001	0.817, *p* < 0.0001

In the high-concentration group (>22 μg/mL, n = 4), the corrected SVPS results showed MPE of −5.40%, RMSE of 7.31%, and a mean absolute bias of 1.35 μg/mL (range: −2.87 to +0.32 μg/mL). As expected, given the small sample size and elevated concentrations, corrected SVPS in this range should be reserved for initial screening only, with confirmatory LC-MS/MS testing required for definitive clinical decision-making.

## 4 Discussion

The efficacy and safety of OXC therapy rely critically on maintaining plasma concentrations of its active metabolite (MHD), within the therapeutic range. Accurate quantification of MHD is essential for optimizing seizure control and minimizing adverse effects. Consequently, routine TDM of MHD is a standard practice of clinical management (Patsalos et al., 2018; Beydoun et al., 2020; Kroner et al., 2021). Beyond direct clinical application, MHD concentration data also serve important roles in pharmacokinetic research, supporting population pharmacokinetics (PPK) and physiologically based pharmacokinetic (PBPK) modeling (Lin et al., 2019a; Lin et al., 2019b; Chen et al., 2021; He et al., 2022; Sinha et al., 2022), which in turn inform individualized dosing strategies (Rodrigues et al., 2017; Chen et al., 2019; Yang et al., 2023). Although LC-MS/MS is considered the gold standard for MHD quantification due to its high specificity and sensitivity, its use in routine clinical laboratories is often limited by technical complexity, high cost, and extended turnaround times. In contrast, EMIT platforms, such as the SVPS, provide a practical alternative for high-throughput settings, offering advantages in speed, operational simplicity, and cost-effectiveness (Yu et al., 2023; Nakagawa and Wakui, 2024).

To assess the feasibility of using the SVPS for MHD TDM, we performed a comparative analysis with LC-MS/MS. The results demonstrated a systematic overestimation of MHD concentrations by SVPS, consistent with previous reports of immunoassay-based methods for other drugs (Prémaud et al., 2004; Li et al., 2017; Xia et al., 2021; Zhao et al., 2022). The EMIT technique, utilized by the SVPS, is based on the competition between MHD and a glucose-6-phosphate dehydrogenase-labeled antibody (Nayak et al., 2023). The overestimation may be attributed in part to antibody cross-reactivity with structurally related compounds. According to the ARK™ Oxcarbazepine Metabolite Assay package insert, the parent drug oxcarbazepine exhibits 22.2% cross-reactivity (ARK Diagnostics, Inc., 2017). Additionally, carbamazepine and its metabolites (Dihydro–carbamazepine and Carbamazepine-epoxide), which share structural similarities with MHD, also demonstrated measurable cross-reactivity. Earlier studies further support these findings: Kumps, A. et al. reported that OXC and MHD cross-react with the carbamazepine-directed EMIT reagents (Kumps and Mardens, 1986), while Parant, François et al. observed limited but non-negligible cross-reactivity of OXC and MHD in both PETINIA and EMIT 2000 assays (Parant et al., 2003). Collectively, these observations indicate that immunoassays are susceptible to cross-reactivity, leading to inflated MHD concentration values. Therefore, it is essential to explicitly specify the analytical method and platform used in TDM reports to ensure accurate clinical interpretation of results.

Notably, the application of Deming regression correction significantly improved concordance between SVPS and LC-MS/MS by effectively reducing systematic bias. While this adjustment resolved the statistical discordance between the two methods across all ranges, the correlation remained suboptimal at higher concentrations (>22.0 μg/mL). In contrast, no significant differences were observed between corrected SVPS and LC-MS/MS results at MHD concentrations below 22.0 μg/mL, indicating full method interchangeability within this clinically relevant range. Therefore, after Deming-based adjustment, SVPS measurements represent a reliable alternative to LC-MS/MS for supporting TDM decisions in both the low (<12.0 μg/mL) and medium (12.0–22.0 μg/mL) concentration subgroups.

Although uncorrected SVPS values showed a mean overestimation of 13.04%, which could lead to inappropriate dose adjustments, applying the Deming regression equation effectively eliminated this bias and ensured clinical concordance with LC-MS/MS. Looking forward, integration of the Deming regression equation into laboratory information systems could allow automatic adjustment of SVPS results before reporting, thereby enhancing clinical applicability. Nevertheless, LC-MS/MS confirmation would remain necessary in critical scenarios, such as suspected toxicity or complex pharmacokinetic conditions.

This study also has several limitations: first, the use of clinical samples from a single center may restrict the generalizability of our findings. Multi-center studies are needed to validate the broader applicability of the proposed Deming correction model. Second, the sample size, especially within the high-concentration subgroup and the independent validation cohort (n = 30), was relatively small, which may limit the robustness of the regression analysis and bias correction. While the results demonstrated excellent consistency between the calibration and validation sets, future investigations with larger and more diverse patient cohorts are warranted to further evaluate and refine the model’s performance. Third, although cross-reactivity with carbamazepine metabolites was considered, the potential effects of co-administered medications on SVPS accuracy were not systematically evaluated. Further studies should investigate such interferences to better reflect real-world clinical scenarios.

## 5 Conclusion

To our knowledge, this is the first study to evaluate the concordance between the SVPS and LC-MS/MS for MHD quantification. Our results showed that SVPS systematically overestimated MHD concentrations by 13.04% compared to LC-MS/MS, with a clear concentration-dependent bias. We developed and validated concentration-specific Deming regression equations to facilitate conversion between SVPS and LC-MS/MS results. After correction, the SVPS method demonstrated clinically acceptable correlation and concordance with LC-MS/MS in the low and medium concentration ranges (<22.0 μg/mL), though performance remained suboptimal at concentrations exceeding 22.0 μg/mL. These findings support the use of SVPS for routine TDM of MHD within its validated concentration range and provide a valuable reference for standardizing practices across clinical laboratories.

## Data Availability

The datasets generated during the current study are not publicly available due to patient privacy restrictions, but are available from the corresponding author on reasonable request in an anonymized form. Requests to access the datasets should be directed to chenmming@fjmu.edu.cn.
